# Public health round-up

**DOI:** 10.2471/BLT.23.010623

**Published:** 2023-06-01

**Catching up on vaccination**

A World Health Organization (WHO) team comes together during the first round of a 3-day house-to-house polio immunization campaign targeting 1.3 million children in Yemen. In April, WHO and health partners launched a targeted global effort to boost vaccination among children following declines during the coronavirus disease 2019 (COVID-19) pandemic. Over 25 million children missed at least one vaccination in 2021 alone, and outbreaks of several vaccine-preventable diseases are already on the rise.

**Figure:**
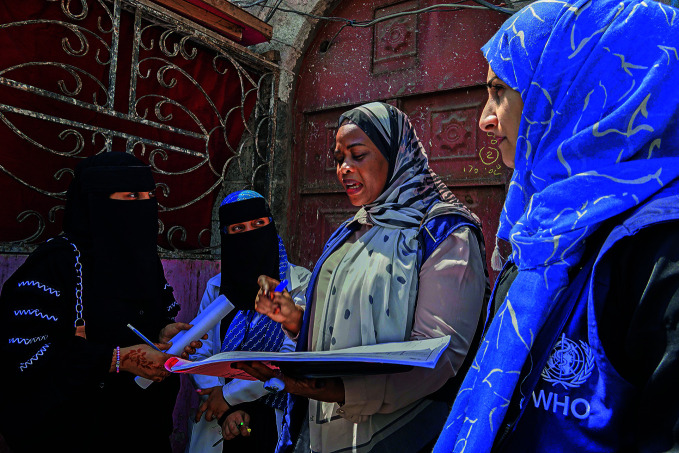
WHO/ Nesma Khan

## COVID-19 and mpox emergencies downgraded

WHO Director-General Tedros Adhanom Ghebreyesus announced that the coronavirus disease 2019 (COVID-19) no longer constitutes a public health emergency of international concern (PHEIC).

The 5 May announcement was made in concurrence with the advice of the International Health Regulations (2005) (IHR) Emergency Committee regarding the pandemic.

Taking into account the decline in COVID-19 deaths, hospitalizations and intensive care unit admissions, and the high levels of population immunity to severe acute respiratory syndrome coronavirus 2 (SARS-CoV-2), the committee advised that it was time to transition to long-term management of the COVID-19 pandemic.

In related news, the mpox emergency was also downgraded from a PHEIC, the Director-General making the announcement on 11 May in concurrence with the advice of the relevant IHR Emergency Committee. 

The IHR Emergency Committee focused on the international spread of poliovirus advised that the PHEIC for polio be extended.

https://bit.ly/3B3xN3h

https://bit.ly/42vMTKU

## Sudan response

With the support of the United Arab Emirates (UAE), WHO flew 30 metric tonnes of urgent medical supplies to Sudan as part of a response to the developing emergency in that country.

A plane carrying supplies for injury treatment, emergency surgeries and essential drugs arrived in Port Sudan Airport on 5 May. The supplies included enough trauma kits, emergency surgical supplies and essential medicines to immediately reach 165 000 people in desperate need of humanitarian aid. Among other support, WHO sent emergency logisticians to ensure that the supplies were immediately distributed to 13 major health facilities in the country.

As of 5 May, WHO was also preparing another 30 metric tonnes of supplies for malaria and noncommunicable diseases, as well supplies of blood, at the WHO global logistics hub in the International Humanitarian City in Dubai, UAE.

https://bit.ly/3VIM12V

## The Big Catch-up

The World Health Organization joined with global and national health partners to launch a targeted global effort to boost vaccination among children, following declines driven by the COVID-19 pandemic.

Announced on 24 April, “The Big Catch-up” aims to reverse the declines in childhood vaccination recorded in over 100 countries since the pandemic began, due to overburdened health services, closed clinics, and disrupted imports and exports of vials, syringes and other medical supplies. Vaccination has also been hampered by social distancing measures restricting travel and access to services.

With over 25 million children missing at least one vaccination in 2021 alone, outbreaks of vaccine-preventable diseases, including measles, diphtheria, polio and yellow fever are already on the rise. The Big Catch-up aims to reverse negative trends.

https://bit.ly/42dSXXT

## Marburg outbreaks

Equatorial Guinea and the United Republic of Tanzania strengthened critical response functions in response to outbreaks of Marburg virus disease which were declared in early February and late March, respectively.

With the support of WHO and partners, in the weeks leading up to 8 May, the health authorities in both countries had mobilized resources for disease surveillance, laboratory activities, clinical case management, and infection prevention and control. They had also initiated risk communication and community engagement, and ensured operations support and logistics.

As of 8 May, Equatorial Guinea had reported that a total of 40 people had been infected (17 laboratory-confirmed and 23 probable cases) with the last confirmed case reported on 20 April. Among the laboratory-confirmed cases, 12 people had died (case fatality rate (CFR) 75%), while all 23 of those assessed to be probably infected had also died.

In the United Republic of Tanzania, a total of nine cases, including eight laboratory-confirmed and one probable case had been reported with the last confirmed case reported on 11 April. Six of the people infected had died (CFR 66.7%). All the cases were reported from Bukoba district, in the Kagera region.

In March, WHO assessed the public health risk posed by the outbreaks to be very high at the national level and high at the sub-regional level. As of 8 May, WHO was continuing to monitor the situation in both countries closely and supporting the response efforts.

https://bit.ly/42GBGqN

## Boosting pandemic preparedness

WHO launched a new initiative designed to help countries prepare for and respond to outbreaks of respiratory pathogens.

Launched on 26 April, the Preparedness and Resilience for Emerging Threats (PRET) initiative incorporates the latest tools and approaches developed during the COVID-19 pandemic and other recent public health emergencies.

PRET represents an evolution in WHO’s approach to pandemic preparedness by focusing on mode of transmission rather than specific disease. WHO will continue to develop and disseminate guidance on specific diseases, as needed.

https://bit.ly/42bUKwM

## Reducing postpartum bleeding

A new approach to treating postpartum haemorrhage known as E-MOTIVE could radically improve women’s chances of surviving childbirth globally. This is according to a study published on 9 May by researchers from WHO and the University of Birmingham in the United Kingdom of Great Britain and Northern Ireland.

Postpartum haemorrhage (defined as the loss of more than 500 mL of blood within 24 hours after birth) is the leading cause of maternal mortality worldwide, affecting an estimated 14 million women each year and resulting in around 70 000 deaths – mostly in low- and middle-income countries.

The study, which involved over 200 000 women in four countries, found that objectively measuring blood loss using a simple, low-cost collection device called a ‘drape’, and bundling together WHO-recommended treatments as opposed to providing them sequentially, resulted in a 60% decrease in severe bleeding.

https://bit.ly/42telJv

## Health systems recovering

Health systems are showing the first signs of recovery in the aftermath of the COVID-19 emergency. This is according to the WHO interim report on the *Fourth round of the global pulse survey on continuity of essential health services during the COVID-19 pandemic: November 2022–January 2023.*

As of early 2023, countries were reporting reduced disruptions in the delivery of routine health services. However, service disruptions persist across countries in all regions and income levels, and across most service delivery settings and tracer service areas.

https://bit.ly/3MriTdB

**Cover photo**

Accompanied by teams from the Ministry of Health and PAHO, a health professional keeps vaccination records at the Surucucu Base Pole in the Yanomami Special Indigenous Health District in northern Brazil.

**Figure:**
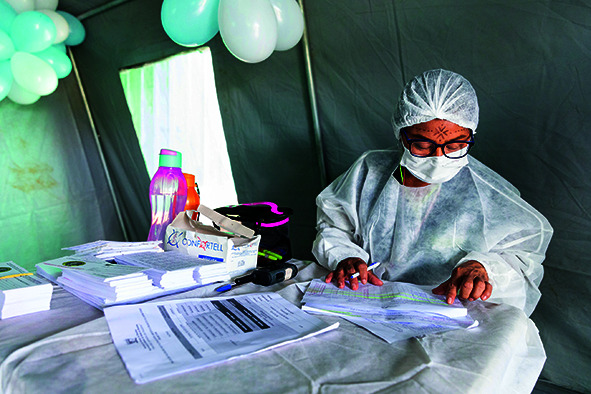
PAHO/WHO-Karina Zambrana

## Researching hand hygiene in health care

WHO released a summary of its first-ever research agenda on hand hygiene in health care. Launched on 5 May, the summary agenda sets out the highest priorities for research needed to improve understanding of the best approaches to improving hand hygiene practices during health care delivery, as well as to generate innovative solutions. 

https://bit.ly/3nUl8Ng

## Health-related SDG progress

The signatories of the Global Action Plan for Healthy Lives and Well-being for All (SDG3 GAP) launched a report assessing four years of collaborative efforts by major multilateral agencies to accelerate country progress towards the health-related sustainable development goals (SDGs).

Launched on 3 May, the report reveals that since its launch in 2019, the SDG3 GAP has enabled the creation of new collaboration structures between the signatory agencies in areas ranging from sustainable financing to primary health care, while at least 67 countries have engaged in one or more of the SDG3 GAP accelerator themes. However, the depth of that engagement varies considerably, while overall progress on the health-related SDGs is lagging.

https://bit.ly/3NIPAEF

## Reducing anaemia

WHO launched its first comprehensive framework on reducing anaemia, and called on countries to accelerate action to halve anaemia prevalence in women of reproductive age by 2025.

Anaemia is a serious global public health problem, affecting 571 million women and 269 million young children worldwide, predominantly in low- and middle-income countries. It increases the risk of infections and death, impairs cognitive performance, and causes extreme fatigue and poor pregnancy outcomes.

https://bit.ly/3BraQY8

**Looking ahead**

13–15 June 2023. Third Global consultation on the health of refugees and migrants. Rabat, Morocco. https://bit.ly/44nmdxi

14–16 June 2023. Small Island Developing States ministerial conference on NCDs and mental health. Bridgetown, Barbados. https://bit.ly/42jYAUJ

27–30 June 2023. Fourth WHO Forum on Alcohol, Drugs and Addictive Behaviours. Geneva, Switzerland. https://bit.ly/3HvqtBe

